# Enhanced Photocatalytic Degradation of the Imidazolinone Herbicide Imazapyr upon UV/Vis Irradiation in the Presence of Ca*_x_*MnO*_y_*-TiO_2_ Hetero-Nanostructures: Degradation Pathways and Reaction Intermediates

**DOI:** 10.3390/nano10050896

**Published:** 2020-05-08

**Authors:** Salma Bougarrani, Preetam K. Sharma, Jeremy W. J. Hamilton, Anukriti Singh, Moisés Canle, Mohammed El Azzouzi, John Anthony Byrne

**Affiliations:** 1 Laboratory of Spectroscopy, Molecular Modelling, Materials, Nanomaterials, Water and Environment, University Med V, Avenue Ibn Battouta, B.P. 1014 Rabat, Morocco; elazzouzim@hotmail.com; 2 NIBEC, Ulster University, Newtownabbey BT37 0QB, UK; jwj.hamilton@ulster.ac.uk (J.W.J.H.); Singh-A3@ulster.ac.uk (A.S.); j.byrne@ulster.ac.uk (J.A.B.); 3 Chemical Reactivity and Photoreactivity Group, Department of Chemistry, Faculty of Sciences & CICA, University of A Coruña, E-15071 A Coruña, Spain

**Keywords:** persistent organic pollutants, photocatalysis, TiO_2_, birnessite, water remediation, hydroxyl radicals, degradation pathways, photoproducts

## Abstract

The determination of reaction pathways and identification of products of pollutants degradation is central to photocatalytic environmental remediation. This work focuses on the photocatalytic degradation of the herbicide Imazapyr (2-(4-methyl-5-oxo-4-propan-2-yl-1H-imidazol-2-yl) pyridine-3-carboxylic acid) under UV-Vis and visible-only irradiation of aqueous suspensions of Ca*_x_*MnO*_y_*-TiO_2_, and on the identification of the corresponding degradation pathways and reaction intermediates. Ca*_x_*MnO*_y_*-TiO_2_ was formed by mixing Ca*_x_*MnO*_y_* and TiO_2_ by mechanical grinding followed by annealing at 500 °C. A complete structural characterization of Ca*_x_*MnO*_y_*-TiO_2_ was carried out. The photocatalytic activity of the hetero-nanostructures was determined using phenol and Imazapyr herbicide as model pollutants in a stirred tank reactor under UV-Vis and visible-only irradiation. Using equivalent loadings, Ca*_x_*MnO*_y_*-TiO_2_ showed a higher rate (10.6 μM·h^−1^) as compared to unmodified TiO_2_ (7.4 μM·h^−1^) for Imazapyr degradation under UV-Vis irradiation. The mineralization rate was 4.07 µM·h^−1^ for Ca*_x_*MnO*_y_*-TiO_2_ and 1.21 μM·h^−1^ for TiO_2_. In the Ca*_x_*MnO*_y_*-TiO_2_ system, the concentration of intermediate products reached a maximum at 180 min of irradiation that then decreased to a half in 120 min. For unmodified TiO_2_, the intermediates continuously increased with irradiation time with no decrease observed in their concentration. The enhanced efficiency of the Ca*_x_*MnO*_y_*-TiO_2_ for the complete degradation of the Imazapyr and intermediates is attributed to an increased adsorption of polar species on the surface of Ca*_x_*MnO*_y_*. Based on LC-MS, photocatalytic degradation pathways for Imazapyr under UV-Vis irradiation have been proposed. Some photocatalytic degradation was obtained under visible-only irradiation for Ca*_x_*MnO*_y_*-TiO_2_. Hydroxyl radicals were found to be main reactive oxygen species responsible for the photocatalytic degradation through radical scavenger investigations.

## 1. Introduction

Persistent organic pollutants (POPs) such as pesticides in the environment are of global concern [1,2]. POPs such as pesticides can be highly toxic to the environment due to their long half-life, stability, and ability of long-range transportation [3]. They can also produce hazardous by-products in a variety of processes: oxidation, hydrolysis, photolysis or other transformations in water [4,5].

New approaches are needed for the treatment of polluted water to expand the range of water sources that can be recycled or re-used, thus contributing towards a circular economy of water. To promote the preservation of long-term water resources, on-site reuse of treated wastewater for agricultural and industrial activities may be the most appropriate technology to ensure availability and sustainable water management in water-deprived regions. Different treatment processes have been explored to reduce or eliminate POPs present in water and to limit health effects due to the exposure to these toxic chemicals by consuming contaminated water [1,6].

Most traditional water treatment methods, including physicochemical treatment and biological degradation, transfer the pollutant from hydrosphere to other environmental compartments [7]. Advanced oxidation processes (AOPs) have the potential to degrade POPs and may lead to complete mineralization (provided treatment is applied for long enough) [8,9].

Among AOPs, heterogeneous photocatalysis using TiO_2_ has been widely investigated for the degradation of POPs in water [8,9,10]. Photocatalysis offers advantages including low cost due to the use of catalytic materials, no consumable chemicals and the potential use of the abundant and renewable solar photons. Although photocatalysis shows a great potential for the degradation of POPs, the solar efficiency is normally poor. With typical photocatalysts such as TiO_2_, the band gap (3.2 eV for anatase) is limited to the UV domain, which accounts for less than 4% of the entire solar spectra thus limiting its ability of utilizing sunlight photons [11]. The poor selectivity of TiO_2_ photocatalysis to eliminate the low-level of POPs in contaminated waters with the presence of other organic material can be considered another disadvantage [12,13].

In order to design highly active photocatalyst systems, researchers have attempted to improve solar efficiency of photocatalyst materials through modification with other photocatalytic or electrocatalytic materials such as RuO_2_, Fe_2_O_3_, ZnO and V_2_O_5_ [14,15]. Surface modification of TiO_2_ can also increase activity through the modification of surface adsorption properties leading to differing selectivity, or changes in recombination due to the development of semiconductor hetero-junctions [15,16].

Two-dimensional (2D) materials are known to have a high surface area and faster charge transport which helps in the separation of the photogenerated charges [15,17]. Most 2D materials are either carbon allotropes, nitrides or metal sulphides [18]. Carbon-based nanomaterials have been used as the co-catalysts but due to their non-existent/limited band-gap or HOMO-LUMO gap, the heterostructures are rarely reported [18,19]. Transition metal dichalcogenides and nitrides are semiconductors whose band-gaps are such that they can be used for photocatalytic reactive oxygen species (ROS) generation applications [5,20]. However, these structures are not very stable. Both nitrides and sulphides are known to degrade in the aqueous media, where any ROS generated attack the photocatalytic material instead of the pollutant or the material undergoes photocorrosion [18,21]. One of the reasons of photocatalytic candidature of metal oxides are their relative stability, same way stable 2D metal oxides can be useful in photocatalysis [18,22]. Layered birnessite material Ca*_x_*MnO*_y_* has attracted a lot of attention due its potential as an oxidation catalyst, with different adsorption properties to TiO_2_ and an narrow band gap of 2.20–2.63 eV, [23,24]. It is reported to have excellent electron transport properties, and may serve as a sensitizer and electron-accepting co-catalyst in MnO*_y_*/TiO_2_ heterostructured material [25,26]. 

Devi et al. reported Mn-TiO_2_ enhanced absorption in the visible region and therefore higher photocatalytic activity under solar irradiation [27]. They attributed such an improvement in activity to the synergistic effect of mixed anatase-rutile phase, smaller crystallite size, and partially filled electronic configuration of Mn^2+^, which could serve as electron and hole shallow traps.

Although it has been shown previously that MnO*_y_* based semiconductors exhibited enhanced photocatalytic performance with respect to the pure photocatalyst alone, some problems still hinder further application of these nanocomposites [28]. To improve on this, different metal ions intercalated into MnO*_y_* (birnessite structure) has been investigated by surface modification with TiO_2_. Recently, Lucht et al. have shown that birnessite structures containing Sr, Al, B, and Ca have good light harvesting ability, and Ca-birnessite type is predicted to be the most suitable candidate for water splitting due its suitable direct band gap for light *c*apturing [29]. Additionally, birnessite readily participates in cation-exchange and oxidation-reduction reactions [30].

In this work, Ca*_x_*MnO*_y_*-TiO_2_ photocatalysts were fabricated and characterised for various physicochemical properties. The materials were tested for the photocatalytic degradation of phenol (as a model POP), and imazapyr (2-(4-methyl-5-oxo-4-propan-2-yl-1H-imidazol-2-yl) pyridine-3-carboxylic acid, an imidazolinone family herbicide) in aqueous suspension and intermediate products were investigated by high-performance liquid chromatography, mass spectrometry and total organic carbon analysis. Also, the influence of Ca*_x_*MnO*_y_* on the photocatalytic activity for Imazapyr degradation has been systematically elucidated. Radical scavenging investigations were carried out to identify the reactive oxygen species involved in the process. The photocatalytic activity and intermediate products for Imazapyr degradation may provide new insight for the use of birnessite materials for photocatalytic water or wastewater treatment.

## 2. Materials and Methods

### 2.1. Reagents

**Figure 1:**
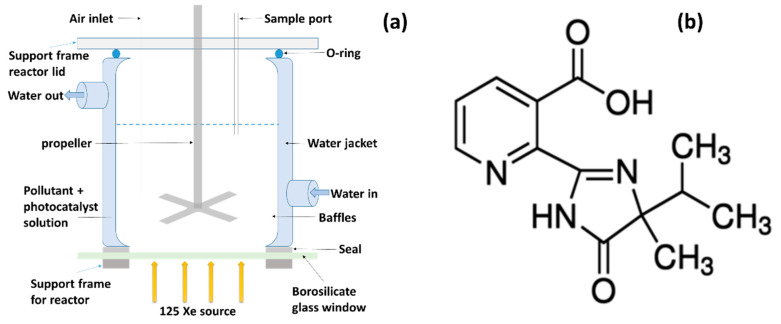
(**a**) Schematic representation of a stirred rank reactor and (**b**) chemical structure of imazapyr herbicide.

TiO_2_ Hombikat UV100 nanoparticles were obtained from Sachtleben Chemie (Duisburg, Germany) and TiO_2_ P25 was obtained from Evonik Aeroxide. Imazapyr herbicide (99% structure shown in Figure 1b), hydrochloric acid, calcium sulphate, sodium hydroxide, potassium permanganate, manganese chloride tetrahydrate, potassium chloride, phenol and various solvents were purchased from Sigma Aldrich UK and used without further purification. Deionized water was obtained from an ELGA Purelab DI water unit (available in NIBEC).

### 2.2. Preparation of the Photocatalyst

Calcium birnessite (Ca*_x_*MnO*_y_*) was synthesized trough a precipitation method described by Frey et al. [13]. In brief, 3.92 g (16 mmol) MnCl_2_·4H_2_O was added to 14.4 g (250 mmol) KOH in 30 mL H_2_O under vigorous stirring. 950 mg (6 mmol) KMnO_4_ in 100 mL H_2_O was then added to the reaction mixture. After 3 h of reaction, the solution was filtered through a Whatman glass fibre filter, followed by drying in oven at 60 °C. Ca*_x_*MnO*_y_* with a variety of Mn to Ca ratios were prepared and the ratio of 1.6 was used for the further investigation due previous investigations towards Imazapyr degradation [25]. Ca*_x_*MnO*_y_* powder (5 wt.%) was mixed with TiO_2_ (Hombikat UV-100) by mechanical grinding followed by annealing under vacuum at 500 °C (ramp 2 °C·min^−1^ up rate and 1 °C·min^−1^ down rate) for 24 h to obtain Ca*_x_*MnO*_y_*-TiO_2_ [25].

### 2.3. Catalyst Characterization

Powder X-ray diffraction (XRD) patterns recorded using an Rigaku-Dmax 2500 instrument with monochromatic Cu K_α_ radiation (0.15405 nm, 40 kV, 100 mA) in the range 10–100°. The zeta potentials of the synthesized catalysts were determined in aqueous solution at different pH values, using a Malvern Zetasizer Nano ZS model. X-ray photoelectron spectra (XPS) of the catalysts were recorded using a Kratos Axis Ultra system. Wide-energy survey scans and high-resolution scans were performed at pass energies of 160 eV and 20 eV, respectively. The obtained XPS spectra were analysed using CasaXPS software (version 2.3.17PR1.1). The calibration of the energy positions was done following normalization of the shifting C 1s peak to 284.8 eV. The XPS peaks were fitted using mixed Gaussian-Lorentzian (GL30) function on a Shirley background. 

High resolution transmission electron microscopy (HRTEM) and selected area electron diffraction (SAED) measurements were performed on JEOL JEM-2100F TEM instrument at 200 kV electron acceleration voltage. Samples for TEM analysis were prepared by the sonication of catalysts in ethanol followed by dip coating or drop-casting on the 3 mm TEM grids. Scanning transmission electron microscopy (STEM) measurements were performed on the same TEM instrument in STEM mode using JEOL high-angle annular dark-field (HAADF) detector at 1.2 nm spot size. The energy dispersive X-ray spectroscopic signals were collected using Oxford instruments X-Max 80 mm^2^ detector coupled with the TEM.

### 2.4. Photocatalytic Degradation of Pollutants

The photocatalytic degradation of pollutants was performed in the stirred tank photocatalytic reactor (STR) shown in Figure 1a which has been previously reported [31]. The reactor consists of a water-jacketed walled vessel with a stainless-steel propeller and baffle to ensure good mixing and mass transfer. A xenon source (125 W) was used as UV-Vis irradiation source (average emission spectrum in Figure S1). A 410-nm UV cut-off filter was used for visible only irradiation of the reactor. The light intensity entering the reactor was measured at different areas across the sample window using a calibrated radiometer (Jobin Yvon Gemini 180). The average UV intensity (280–400 nm) was determined to be 12.3 W·m^−2^. Water circulation was maintained throughout the photocatalytic experiments to maintain a constant reaction temperature of 25 °C and air was purged before and during the experiments using an aquarium pump.

### 2.5. Radical Scavenging Investigation

Sacrificial reagents with high affinity toward reactive oxygen species (ROS) have been used to investigate their contribution, providing insights into the photocatalytic mechanism [17]. The nature of the ROS generated during Imazapyr photodegradation in the presence of unmodified TiO_2_, Ca*_x_*MnO*_y_*-TiO_2_, and Ca*_x_*MnO*_y_* were established by using specific ROS scavengers. 

*p*-nitrosodimethylaniline (RNO) dye was used as probe compound and a spin trap for the detection of hydroxyl radicals (HO^•^). The bleaching of RNO has been reported to be very selective to oxidation by HO^•^. Superoxide radical (O_2_^•−^) detection was investigated using (1,4)-benzoquinone (BQ) as a probe. BQ reacts with O_2_^•−^ leading to the formation of a semiquinone, which can be detected by its absorption peak at 430 nm [15,17].

For HO^•^ detection, the photocatalytic materials were suspended in a solution of RNO (17 µM) and irradiated with UV–Vis and visible-only irradiation under continuous stirring. Samples were taken at 2 min intervals, centrifuged, and the optical absorbance was recorded at 440 nm in a UV–Vis spectrometer. Controls were performed in the absence of catalyst to determine any photolytic effect. 

For O_2_^•−^, detection a 300 µM solution of BQ was used. The photocatalytic materials along with Imazapyr were dissolved in the BQ solution. The generated O_2_^•−^ are captured by BQ, and if superoxide is the active ROS, then the rate of pollutant degradation decreases. Unmodified TiO_2_ and Ca*_x_*MnO*_y_*-TiO_2_ were irradiated with UV–Vis light under continuous stirring. Samples were taken at different intervals during 300 min and centrifuged to separate the catalyst before the determination of Imazapyr concentration. Imazapyr concentrations were determined using reverse phase HPLC system equipped with a UV detector (vide infra).

### 2.6. Analytical Methods

#### 2.6.1. High Performance Liquid Chromatography (HPLC)

Imazapyr and phenol concentrations were determined using reverse phase HPLC system equipped with a UV detector (Agilent 1200 series) with Supelco IL (250.0 × 4.6 mm, 5 µm separation column) and Supelco guard column (supelguard C18 (22.0 mm × 4.0 mm, 5 μm). The mobile phase was a mixture of 40% methanol and 60% water adjusted to pH 3.2 by adding formic acid, isocratic elution at a flow rate 1 mL·min^−1^. The injection volume of 20 μL and 250 nm UV detection wavelength were used for Imazapyr. For phenol, 30 μL injection volume and UV detection wavelength of 270 nm was used.

Six different Imazapyr concentrations were used to build a calibration curves (R^2^ value of 0.999). The limits of detection (LOD), and of quantitation (LOQ) were calculated, with values 9.82 ppb (LOD) and 29.47 ppb (LOQ) for phenol and 6.53 ppb (LOD) and 18.62 ppb (LOQ) for Imazapyr.

#### 2.6.2. Mass Spectrometry-Electrospray Ionisation (MS-ESI)

The by-products generated during the photodegradation of Imazapyr were determined by mass spectrometry-electrospray ionization (MS-ESI) recorded on Bruker Esquire 3000 plus instrument. The working parameters of ESI were previously determined by Bougarrani et al. [25].

#### 2.6.3. Total Organic Carbon (TOC) Analysis

TOC analysis was carried out using a Shimadzu 5000A analyser. The TOC analyser measures total carbon (TC) and inorganic carbon (IC) and determines TOC by subtracting the IC contribution from TC. The TC is determined by oxidizing the carbon in a furnace at 680 °C using Pt catalyst. The IC is determined by the acidification of the sample to convert IC to CO_2_. The CO_2_ liberated by both TC and IC analysis was quantitatively determined by IR analysis.

## 3. Results and Discussion

### 3.1. Characterization of Materials

X-ray diffraction peaks of pure TiO_2_ can be assigned to the anatase phase, rutile phase was not detected indicating that samples retained their original phase during calcination and no peaks associated with separated birnessite phases which should appear at about 12° and 25° were detected [32,33]. 

This could be either explained by the fact that the amount of birnessite/MnO_2_ did not exceed 5% in the total composition of Ca*_x_*MnO*_y_*-TiO_2_, or due to the total concentration of amorphous to crystalline material [34]. However, the peaks of Ca*_x_*MnO*_y_*-TiO_2_ composite are much sharper than TiO_2_, which indicate bigger particle size. The Scherrer equation was used to determine the particle size and it was confirmed that crystalline sizes of TiO_2_ and Ca*_x_*MnO*_y_*-TiO_2_ (annealed at 500 °C) are 8.4 and 19.9 nm, respectively. 

Supplementary Figure S2 shows the absorption spectra of Ca*_x_*MnO*_y_*-TiO_2_. It was confirmed from spectra that pure TiO_2_ shows its characteristic sharp absorption edge at 387 nm, which is consistent with the band gap of anatase TiO_2_ (ca. 3.2 eV) [35]. Ca*_x_*MnO*_y_*-TiO_2_ by contrast shows a broad adsorption across the visible range due to surface modification. 

**Figure 2:**
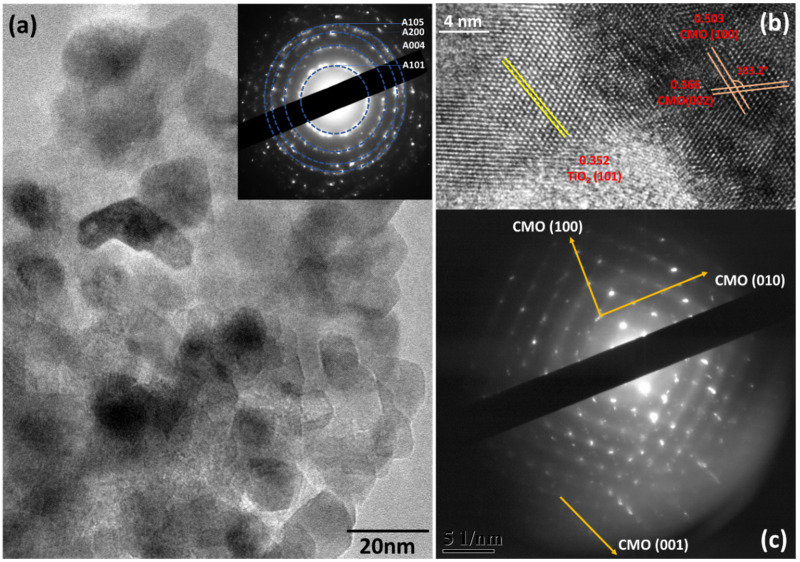
(**a**) TEM and (**inset**, **a**) corresponding SAED from the Ca_x_MnO_y_-TiO_2_ samples. (**b**) HRTEM, and (**c**) SAED of Ca*_x_*MnO*_y_*-TiO_2_.

TEM images of Ca*_x_*MnO*_y_*-TiO_2_ are shown in Figure 2. The different TiO_2_ particles fuse together as shown in Figure 2a. The inset in 2a shows the SAED pattern from the area. From SAED, various anatase planes (101, 004, 200, and 105) were identified and labelled. Figure 2b shows the HRTEM from the sample. As shown in the figure, the lattice spacing of 0.352 nm corresponds to anatase (101) plane. Birnessite is a manganese oxide mineral with a layered structure [30]. The lattice spacings of 0.503 and 0.366 nm correspond to birnessite planes (100) and (002), respectively [36]. The angle of 103° between (100) and (002) further confirms the assigned planes. Figure 2c shows the SAED from Ca*_x_*MnO*_y_*-TiO_2_. The image was captured at a lower camera length as compared to inset in Figure 2a to reveal the patterns from Ca*_x_*MnO*_y_*. As shown, Ca*_x_*MnO*_y_* is highly crystalline. The (100) and (010) planes corresponding to Ca*_x_*MnO*_y_* were identified. Diffraction pattern with spacing corresponding to (001) plane were also identified. Looking at the relative position of (001) diffraction pattern, the signals should be coming from a different set of Ca*_x_*MnO*_y_* layers.

**Figure 3:**
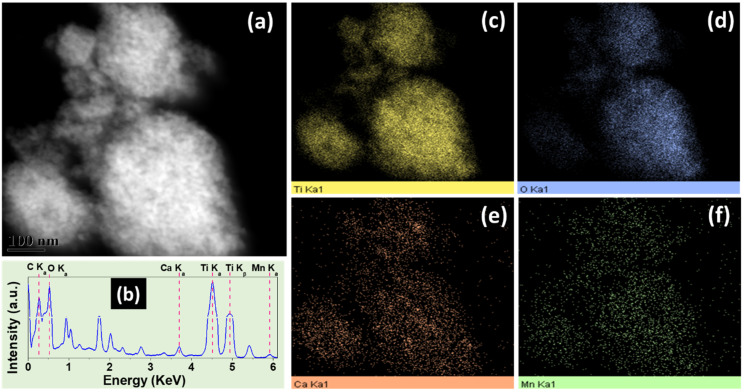
(**a**) HAADF-STEM image and corresponding (**b**) map-sum EDX spectra. The elemental maps corresponding to (**c**) Ti, (**d**) O, (**e**) Ca and (**f**) Mn are also shown.

Figure 3a shows the HAADF-STEM image from the Ca*_x_*MnO*_y_*-TiO_2_ sample. The energy dispersive Xray analysis (EDX) spectra corresponding to the area is shown in Figure 3b. As shown in figure, the peaks corresponding to Ca, Mn, Ti and O were obtained. The peak intensities of Ca and Mn is much lower than Ti due to Ca*_x_*MnO*_y_* being only 5% of TiO_2_. The EDX maps corresponding to the elements of interest, namely Ti, O, Ca, and Mn, are shown in Figure 3c–f, respectively. As expected, the signals from Mn and Ca are much lower than those from Ti. However, the elemental maps overlap well, indicating that Ca*_x_*MnO*_y_* is well distributed on TiO_2_ surfaces.

**Figure 4:**
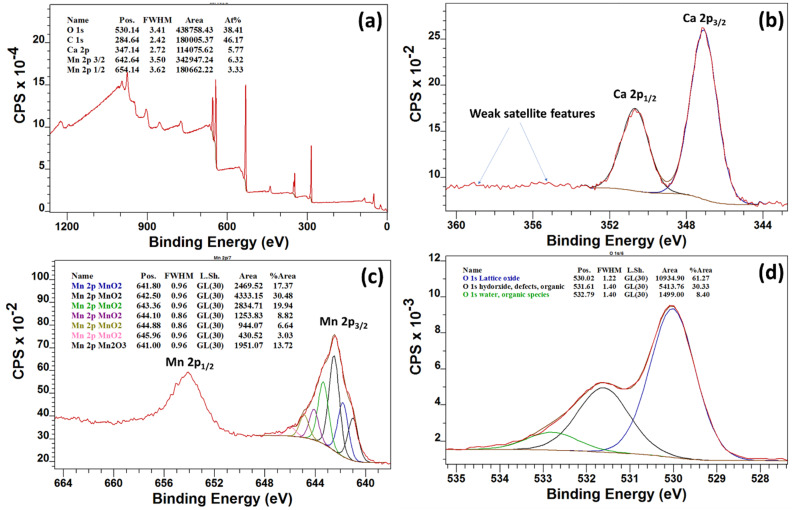
(**a**) Survey, (**b**) Ca 2p, (**c**) Mn 2p and (**d**) O 1s of Ca*_x_*MnO*_y_* samples.

The chemical composition of Ca*_x_*MnO*_y_*-TiO_2_ was further characterized by X-ray photoelectron spectroscopy (XPS). As Ca*_x_*MnO*_y_* is only 5% of TiO_2_ (by weight) XPS analysis of Ca*_x_*MnO*_y_* alone was carried out to determine the elemental composition (Figure 4). The survey scan (Figure 4a) indicates that the material has Ca, Mn and O along with traces of carbon. The ratio of Mn to Ca is 1.67 which is in agreement with the precursor ratio of 1.6. The high-resolution scans for Ca 2p, Mn 2p and O 1s are shown in Figure 4b–d. The binding energy position of Ca 2p_3/2_ is 347.2 and Mn 2p_3/2_ is 642.5 eV which are corresponding to the oxides [37,38,39]. As shown in Figure 4c, Mn is mainly in the Mn^4+^ state with some (ca. 13.7%) Mn^3+^.

The XPS analysis of Ca*_x_*MnO*_y_*-TiO_2_ is shown in Figure S4. The wide-energy survey scan in Figure S4a shows the peaks corresponding to C (adventitious), Ti, Ca, Mn, and O. The Ca*_x_*MnO*_y_* to TiO_2_ ratio is 3.3 from the survey spectra. The high-resolution scans for Ti 2p, O 1s, Ca 2p, and Mn 2p regions are shown in Supplementary Figure S4b–e. The binding energy position of Ca, Mn, and Ti are consistent with the oxides position in the literature [23].

**Figure 5:**
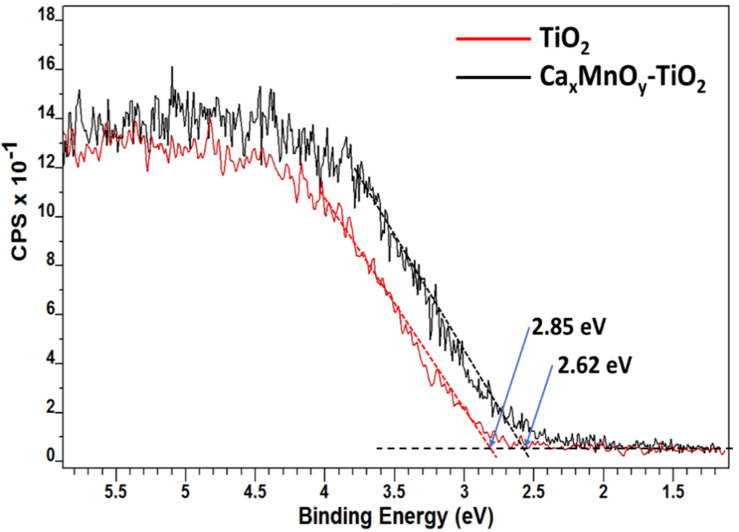
Valence Band XPS of TiO_2_ and Ca*_x_*MnO*_y_*-TiO_2_.

The valence band (VB) XPS was utilised to determine the VB edge for the materials as shown in Figure 5. The valence band edge position vs. Fermi level for Ca*_x_*MnO*_y_*-TiO_2_ and TiO_2_ were 2.62 and 2.85 eV, respectively. The shift of the VB edge to a lower value, by around 0.2 eV, indicates a band-gap narrowing which is consistent with the UV-Vis absorption spectra recorded by the authors previously [25].

Ca*_x_*MnO*_y_* layers encapsulate TiO_2_ particles, resulting in an effective separation of charge-carrier traps and leading to an efficient e^−/^h^+^ separation, which result in a lower photoluminescence intensity as compared to unmodified TiO_2_ [40,41]. The PL spectra from the materials is shown in our previous publication [25]. On the other hand, lower degradation efficiency was observed when higher concentration of Ca*_x_*MnO*_y_* was used in the synthesis of the composite, this could hypothetically be explained by faster recombination caused by a surplus of Ca*_x_*MnO*_y_* which can act as a recombination centre for e^−^ and h^+^, or due to the shielding effects [42].

**Figure 6:**
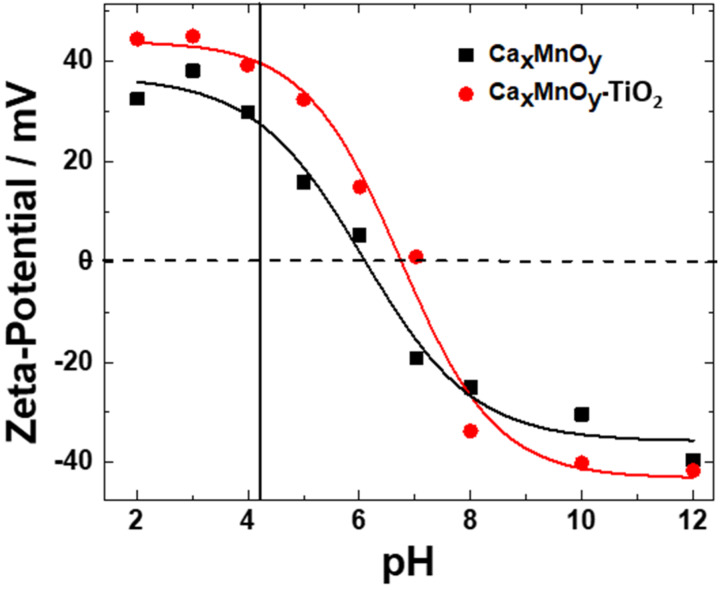
Zeta potentials at different pH in 1 mM KCl.

To understand better the pollutant photocatalyst surface interactions related to photodegradation of Imazapyr, the surface electrostatic charges of the suspended catalysts were investigated. Zeta potential values and isoelectric point of TiO_2_ and Ca*_x_*MnO*_y_*-TiO_2_ were measured in aqueous solution with different pH values. The obtained results are presented in Figure 6 and Table 1. Fitting the observed curves to the expected sigmoidal behaviour, a small shift is observed in the point of zero charge between materials, pH_ps_ of 6.1 for TiO_2_ and pH_pzc_ of 6.7 for Ca*_x_*MnO*_y_*-TiO_2_. Atitar et al. have reported that Imazapyr shows 5 pKa corresponding to five different ionic equilibria [43]. The adsorption of Imazapyr on the surface of catalyst is accelerated with decreasing the pH of the solution, resulting in stronger and more stable electrostatic interactions, which is expected to enhance the photocatalytic degradation rate of Imazapyr [44]. Carrier et al. have reported similar results for photocatalytic degradation of Imazapyr [45].

The extent of surface adsorption of pollutants is key to photocatalytic activity [43,45]. The surface charge affects the adsorption of charged pollutants and this electrostatic interaction is strongly affected by pH [3,46]. Comparing unmodified TiO_2_ and Ca*_x_*MnO*_y_*-TiO_2_ composite, the improvement of the photocatalytic activity in the presence of Ca*_x_*MnO*_y_* for pH 4.1 ± 0.1 could be explained by the stronger electrostatic attraction between negatively charged Imazapyr and Ca*_x_*MnO*_y_*-TiO_2_, more positively charged (37.73 mV) as compared to TiO_2_ (26.3 mV).

### 3.2. Photocatalytic Activity of Ca_x_MnO_y_-TiO_2_

The photocatalytic activity of TiO_2_–Hombikat UV100 and Ca*_x_*MnO*_y_*-TiO_2_ in aqueous medium, under UV-Vis and visible-only irradiation, was assessed using phenol and Imazapyr as model compounds. The photocatalytic activity was compared to commercial TiO_2_ P25. From here onwards, TiO_2_ refers to Hombikat UV100 and P25 is mentioned whenever used for comparison.

The kinetics of heterogeneous photocatalysis processes were treated using a modified Langmuir-Hinshelwood kinetic model, as in Equation (1):

(1) $$r=k_{LH}\cdot \Theta =k_{LH}\cdot \frac{K_{LH}\cdot C}{1+K_{LH}\cdot C}$$

where *C* is the concentration of the aromatic compound in the reaction medium, once the adsorption equilibrium has been established, *k_LH_* (mol·s*^−^*^1^·cm^−2^) is an apparent kinetic rate constant per unit of surface area, and Θ (cm^2^) accounts for the coverage of catalyst surface by phenolic compound. *K_LH_* is the Langmuir-Hinshelwood adsorption constant. Assuming *K_LH_·C* <<*1*, Equation (1) reduces to a first order kinetic model Equation (2):

(2) $$r=k_{app}\cdot C$$

where

*k_app_* ≈ *k_LH_*·*K_LH_* (3)

is the limiting apparent pseudo-first order kinetic rate constant when *K_LH_·C* << 1.

Since heterogeneous photocatalysis depends on the initial concentration of the organic substrate, C_0_, and this was kept constant, fitting the obtained photodegradation curves to pseudo-first order kinetics, according to the described modified Langmuir–Hinshelwood model above, allowed to obtain the corresponding rate constants.

**Figure 7:**
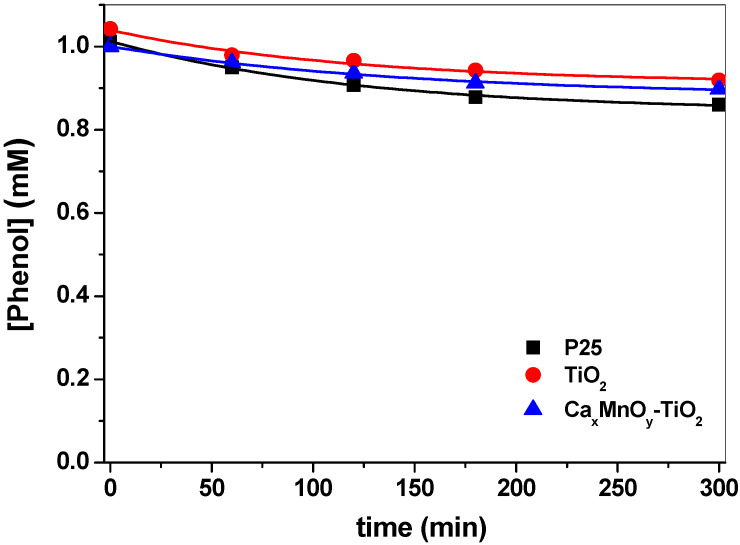
Change in phenol concentration over time upon photocatalysis under UV-Vis irradiation in the presence of TiO_2_-P25, TiO_2_-Hombikat UV100 and Ca*_x_*MnO*_y_*-TiO_2_.

**Figure 8:**
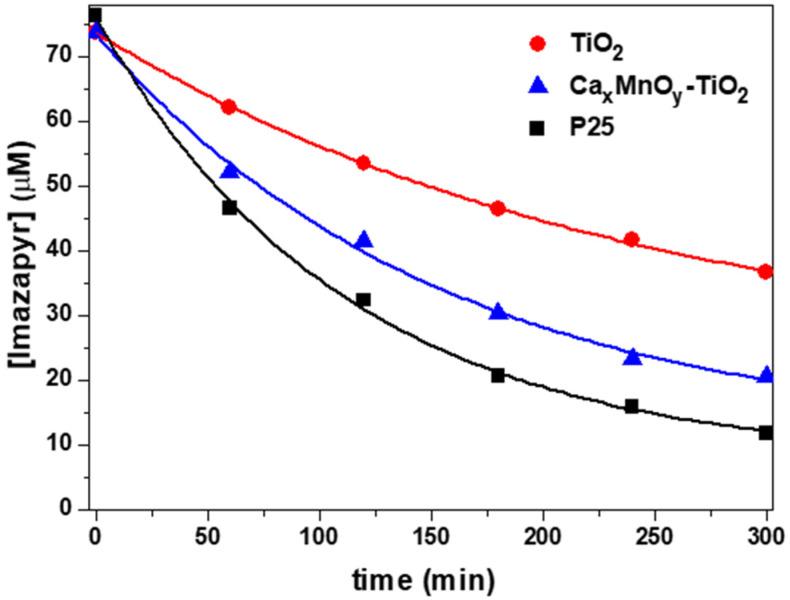
Change in Imazapyr concentration over time for TiO_2_-Hombikat UV 100 and Ca*_x_*MnO*_y_*-TiO_2_ under UV-Vis irradiation, as determined by HPLC monitoring.

As shown in Figure 7 and Figure 8, Ca*_x_*MnO*_y_*-TiO_2_ and TiO_2_-Hombikat UV 100 show a photocatalytic activity under UV-Vis irradiation that is the same within error k_phenol + Ca*x*MnO*y*-TiO2_ = (5.0 ± 0.4)·10^−4^ min^−1^ vs. k_phenol + TiO2_ = (5.2 ± 0.1)·10^−4^ min^−1^. In turn, Ca*_x_*MnO*_y_*-TiO_2_ shows higher photoactivity toward Imazapyr (k_Imazapyr + CaxMnOy-TiO2_ = (4.3 ± 0.2)·10^−3^ min^−1^) as compared to TiO_2_ Hombikat UV 100(k_Imazapyr_ _+ TiO2_ = (2.31 ± 0.09)·10^−3^ min^−1^). Modification of TiO_2_ with Ca*_x_*MnO*_y_* leads to a ca. 2-fold rate enhancement for Imazapyr transformation, with essentially no change for phenol, an effect that may be attributed to the different modes of adsorption of both molecules onto the surface of the catalysts.

P25, one of the most active TiO_2_ formulations, is shown for comparison, with rate constants k_phenol + P25_ = (7.8 ± 0.8)·10^−4^ min^−1^ and k_Imazapyr + P25_ = (6.2 ± 0.3)·10^−3^ min^−1^. In both cases, the process is faster than with unmodified and modified TiO_2_, which may be attributed to a higher efficiency in the generation of active species, possibly associated with a lower e^−^/h^+^ recombination.

Ca*_x_*MnO*_y_*-TiO_2_ was also tested as catalysts of the photo-degradation of Imazapyr under Vis irradiation (λ > 410 nm). The results showed a degradation rate (k_Imazapyr + CaxMnOy-TiO2_ = (3.6 ± 0.3)·10^−4^ min^−1^) that is ca. 8% of that obtained under UV-Vis. No degradation of Imazapyr was observed in the same time span when using TiO_2_-Hombikat UV 100 and TiO_2_-P25.

**Figure 9:**
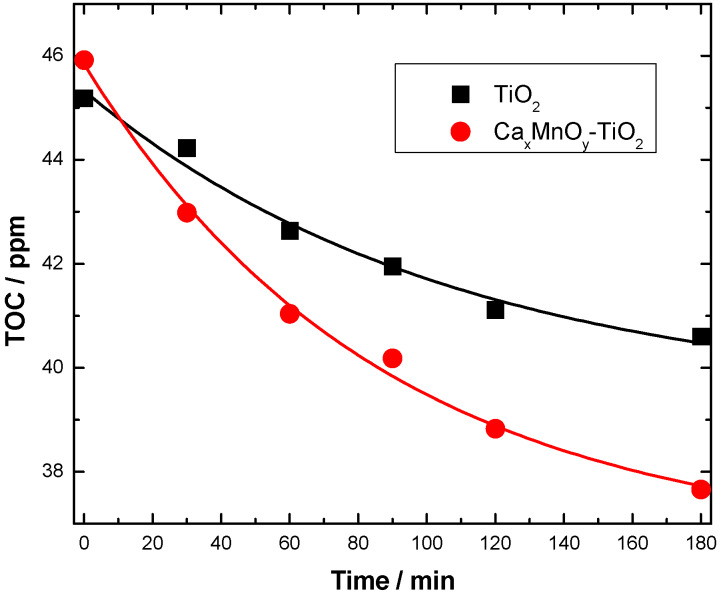
TOC versus time for transformation of Imazapyr under UV-Vis irradiation.

The mineralization of Imazapyr was evaluated by monitoring the changes in TOC as function of time in the reaction systems (Figure 9). Upon comparison, the observed mineralization rate constants are k_TOC + CaxMnOy-TiO2_ = (1.5 ± 0.2)·10^−3^ min^−1^ and k_TOC + TiO2_ = (8.6 ± 0.9)·10^−4^ min^−1^. Both rate constants are ca. 35–40% of those obtained for Imazapyr, evidencing that its transformation intermediates show a lower reactivity. From these values, the lifetimes for complete mineralization would be above 11 h for Ca*_x_*MnO*_y_*-TiO_2_ and 19 h for TiO_2_ Hombikat UV 100, respectively, showing the advantage of the heterostructure.

#### 3.2.1. Detection of ROS under UV–Vis and Vis Irradiation

**Figure 10:**
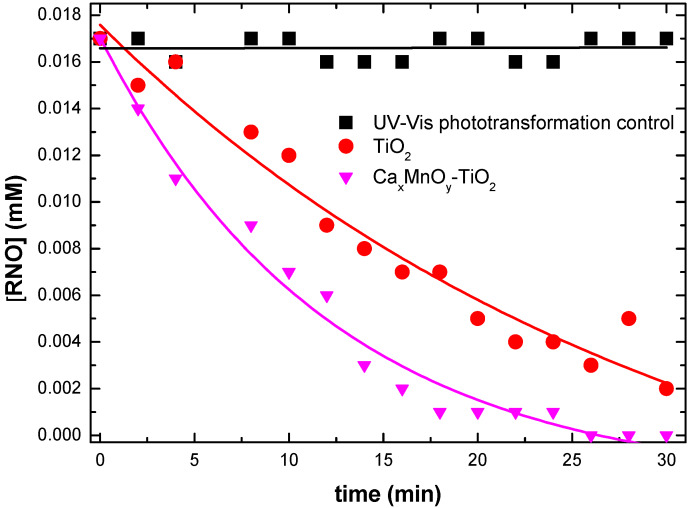
HO^•^ detection by means of RNO bleaching under UV-Vis irradiation for Ca*_x_*MnO*_y_*-TiO_2_, TiO_2_ and transformation control.

HO^•^ production was investigated in the presence of unmodified TiO_2_-Hombikat UV 100, Ca*_x_*MnO*_y_*-TiO_2_, and Ca*_x_*MnO*_y_* suspended in RNO. The solution was irradiated with both UV–Vis and visible-only irradiation. HO^•^ detection under UV–Vis irradiation (Figure 10) in the presence of unmodified TiO_2_ Hombikat UV 100, Ca*_x_*MnO*_y_*-TiO_2_, and Ca*_x_*MnO*_y_* (as a UV-Vis transformation control) was assessed by means of RNO bleaching Equation (4).

RNO+ HO^•^ → RNO•OH (4)

Ca*_x_*MnO*_y_*-TiO_2_ showed higher production of HO^•^ than TiO_2_-Hombikat UV 100, suggesting that the Ca*_x_*MnO*_y_* enhanced the transfer of the photogenerated electrons from the conduction band of TiO_2_ to the conduction band of Ca*_x_*MnO*_y_*, leaving behind positively charged valence band holes to form HO^•^ radicals. Thus, a reduced rate of charge carrier recombination is achieved along with an increased photocatalytic oxidation of RNO by scavenging of HO^•^ radicals produced in water. In contrast, Ca*_x_*MnO*_y_* alone did not produce HO^•^ radicals.

**Figure 11:**
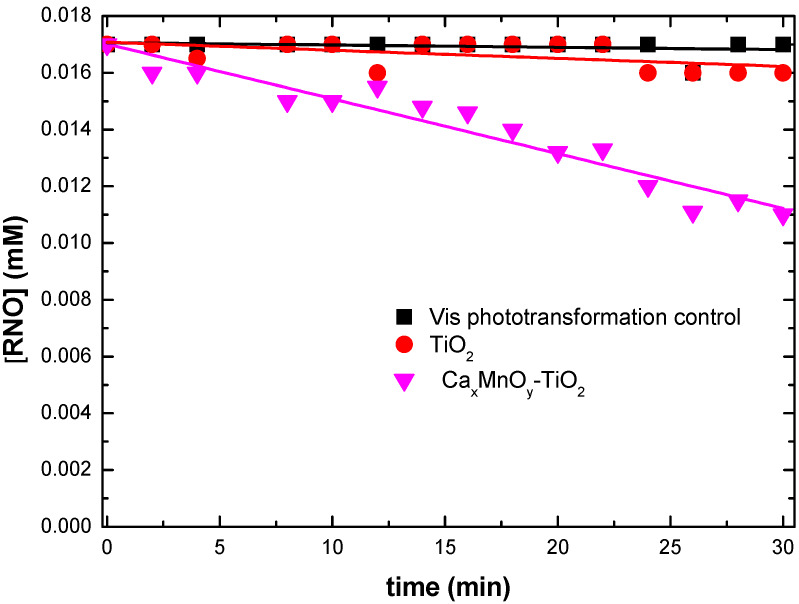
HO^•^ detection by means of RNO bleaching under visible-only irradiation for Ca*_x_*MnO*_y_*-TiO_2_, TiO_2_ and visible-only transformation control. This was achieved by using a cut off filter to block the UV radiation with λ < 410 nm.

The RNO bleaching experiment was also performed under visible-only irradiation (cut off λ < 410 nm). As shown in Figure 11, unmodified TiO_2_, and Ca*_x_*MnO*_y_* did not produce HO^•^ radicals under visible-only irradiation. In turn, Ca*_x_*MnO*_y_*-TiO_2_ produced a significant amount of HO^•^ radicals. These results show that surface modification of TiO_2_ with Ca*_x_*MnO*_y_* lead to increased visible-light photocatalytic activity, which is important for more efficienct solar-driven applications of photocatalysis.

**Figure 12:**
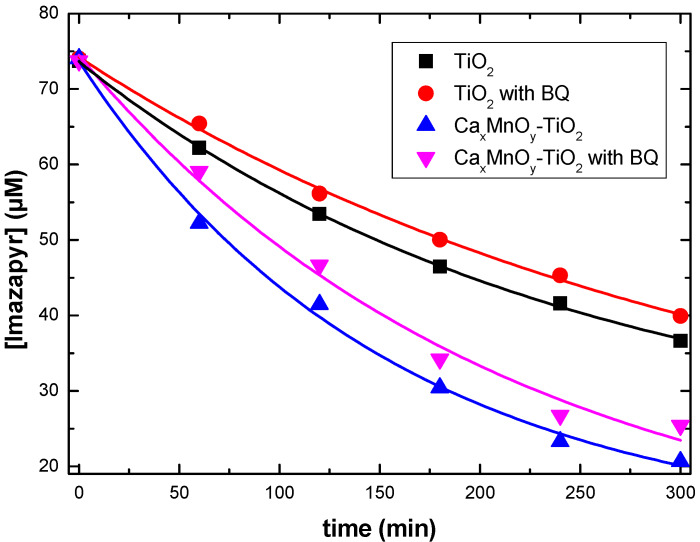
Imazapyr degradation in the presence of both catalyst in the presence and absence of BQ.

BQ was used as a probe for O_2_^•−^ formation under UV–Vis irradiation. Figure 12 shows very small differences in the presence and absence of BQ both for unmodified TiO_2_ (9% variation) and Ca*_x_*MnO*_y_*-TiO_2_ (8% variation). Such small differences suggest an almost negligible contribution from O_2_^•−^ to the process. As the transformation is much more relevant than the contribution of O_2_^•−^ accounts for, it can be concluded that the main ROS generated during photocatalysis with these materials are HO^•^ radicals.

#### 3.2.2. Investigation of the Reaction Products and Reaction Mechanism

**Figure 13:**
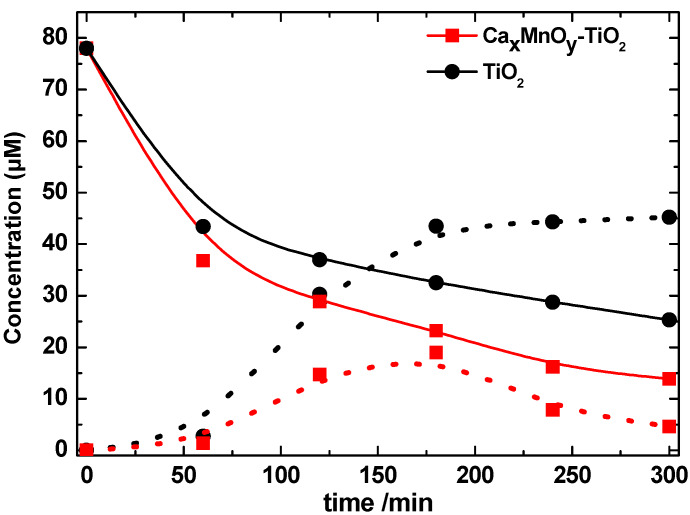
Evolution of concentrations of Imazapyr (solid line) and total intermediates (dots) upon photocatalysis onto Ca*_x_*MnO*_y_*-TiO_2_ (I2) and TiO_2_ (I1), as determined by MS-ESI.

**Figure 14:**
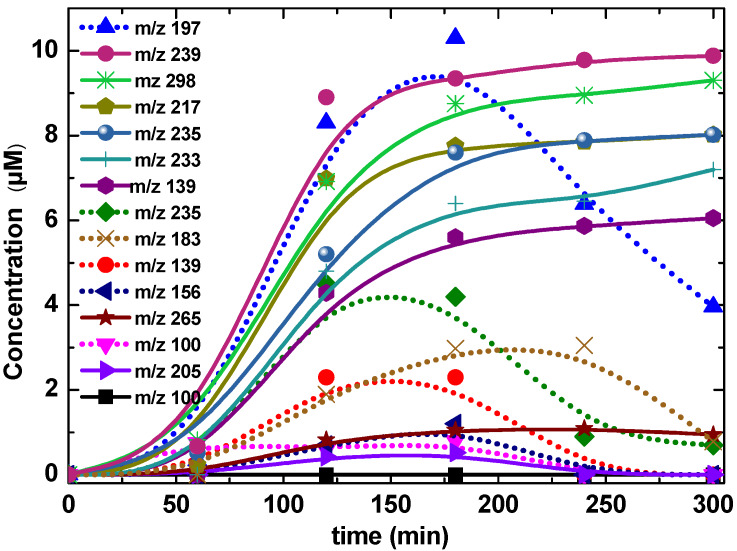
Change in concentration vs. time for various intermediates (represented by their corresponding m/z) of the photocatalytic Imazapyr degradation, determined by the MS-ESI.

To investigate the degradation profiles of intermediates produced on catalysts Ca_x_MnO_y_-TiO_2_ (dots) and TiO_2_ (solid line) as shown in Figure 13 and Figure 14, mass spectroscopy electrospray ionisation (MS-ESI) experiments were performed. ESI was used for the detection of both positively and negatively charged intermediates of the photocatalytic degradation by coupling a quadrupole ion trap mass spectrometer with continuous polarity switching. From the mass spectrometric fragmentation of the most intense ions, intermediates were identified after a 5-h degradation experiment. The major final products in the presence of TiO_2_ and Ca*_x_*MnO*_y_*-TiO_2_ detected are compared to the corresponding peak areas, as monitored by MS-ESI, and listed in Table 2.

As shown in Figure 13, not only is Imazapyr more efficiently transformed by Ca*_x_*MnO*_y_*-TiO_2_, but the total concentration of intermediate products generated after 180 min photocatalytic degradation of Imazapyr using Ca*_x_*MnO*_y_*-TiO_2_ (42.2 mM) is lower than when TiO_2_ (75.92 mM) was used. In the presence of Ca*_x_*MnO*_y_*-TiO_2_ the concentration of intermediate products achieved a maximum following 180 min of irradiation before a subsequent decrease of the intermediate concentration. In contrast, with TiO_2_, different intermediate products were observed which continuously increased with irradiation time, but no decrease in their concentration was observed. The differences in TiO_2_ product removal profiles between photocatalysts under study suggest Ca*_x_*MnO*_y_*-TiO_2_ constitutes a better photocatalyst, not only due to the higher degradation rate of the herbicide Imazapyr pollutant, but due to the more efficient degradation of the intermediate products.

The results summarized in Table 2 show that at least nine intermediates, including one detected in the negative mode, were generated in by TiO_2_, while only six intermediates, including four detected in the negative mode, were generated in the presence of Ca*_x_*MnO*_y_*-TiO_2_.

**Figure 15:**
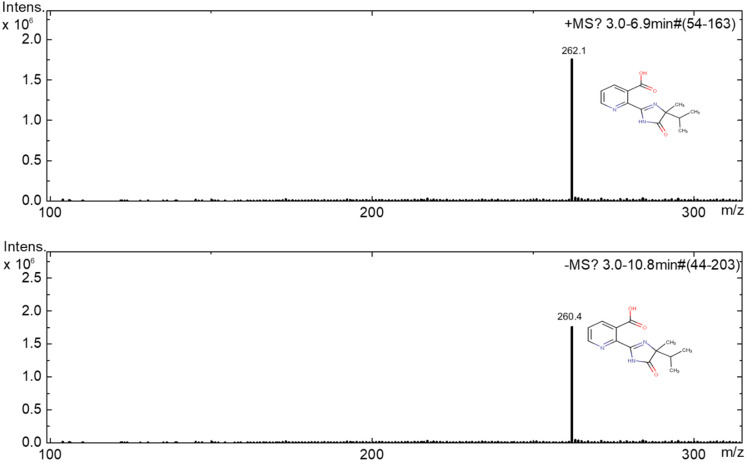
MS (positive and negative) mode of Imazapyr before irradiation.

**Figure 16:**
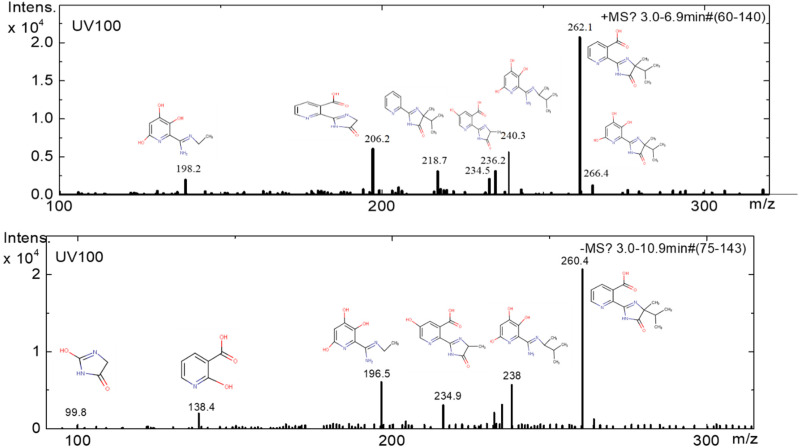
MS (positive and negative) mode of products generated after 300 min of irradiation using TiO_2_ photocatalyst.

**Figure 17:**
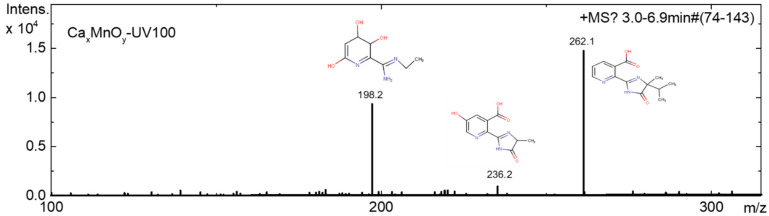
MS (positive and negative) mode of products generated after 300 min of irradiation using Ca*_x_*MnO*_y_*-TiO_2_.

Figure 15, Figure 16 and Figure 17 show the positive and negative mode mass spectra for Imazapyr photocatalysis using both TiO_2_ and Ca*_x_*MnO*_y_*-TiO_2_.

**Figure 18:**
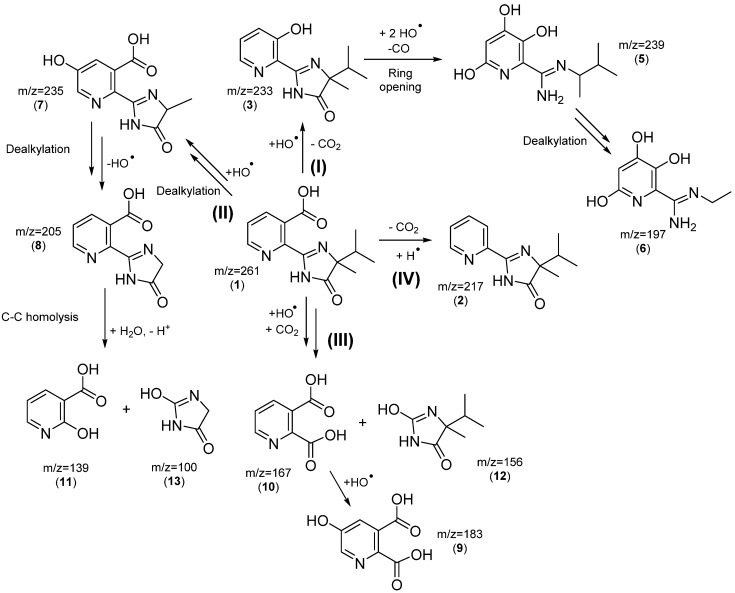
Proposed degradation pathways for Imazapyr.

The structures of intermediates were identified by MS-ESI after 300 min of degradation in the positive and negative mode. The identified intermediates allowed us to propose various competing degradation pathways for degradation of Imazapyr in aqueous solution (Figure 18).

In pathway (I), product (**3**), with m/z = 233, forms by decarboxylation on the pyridine ring initiated by attack of an HO^•^ radical. Product (**5**), with m/z = 239, is attributed to consecutive hydoxylations of the aromatic ring by HO^•^ followed by breakage of the imidazole ring via CO loss and protonation of the nitrogen. Then, the aliphatic chain of the above molecule is transformed through successive demethylations to yield (**6**), with m/z = 197.

In pathway (II), (**7**), with m/z = 235 is formed by hydroxylation and successive demethylations. Additional demethylation on the imidazole ring and dehydroxylation yield (**8**), with m/z = 205. Products (**11**), with m/z = 139 and (**13**), with m/z = 100 are formed by C–C bond scission between the imidazole and pyridine rings.

Pathway III accounts for an alternative HO^•^ attack, leading to bond breaking between the pyridine and imidazole rings, followed by reaction with CO_2_, to yield (**10**), with m/z = 167, and (**12**), with m/z = 156. Hydroxylation of (**10**) yields (**9**), with m/z = 183, detected in ESI(−). This pathway is predominant in the anionic form of Imazapyr, and could explain Imazapyr degradation pathway in the presence of Ca*_x_*MnO*_y_*-TiO_2_.

Compound (**2**), with m/z = 217 is formed in Pathway IV through decarboxylation of the pyrimidinic ring, confirming oxidation with holes h^+^, in addition to HO^•^, as we have recently shown for other compounds possessing carboxylic acid groups. The degradation mechanism involves competition between oxidative decarboxylation of chemisorbed compounds by semiconductor holes, and hydroxyl radical attack on physisorbed substrates [44]. The initial Imazapyr degradation products indicate that Imazapyr adsorbs on the surface of photocatalyst through the carboxylic group [45].

As most of the by-products formed after 300 min of irradiation using Ca*_x_*MnO*_y_*-TiO_2_ catalyst were negatively charged and mainly of small molecular weight, their binding to the catalyst surface would be enhanced and this could increase the degradation process. Fu et al. [1] reported that photodegradation of RhB with two photocatalyst systems occurred via two competitive pathways and have discussed different intermediate products identified in both systems, the enhanced photocatalytic activity of the parent sample for intermediate products was ascribed to the surface modification and the enhancements in the adsorption of the polarised organic reactants.

#### 3.2.3. Recyclability of Ca*_x_*MnO*_y_*-TiO_2_

**Figure 19:**
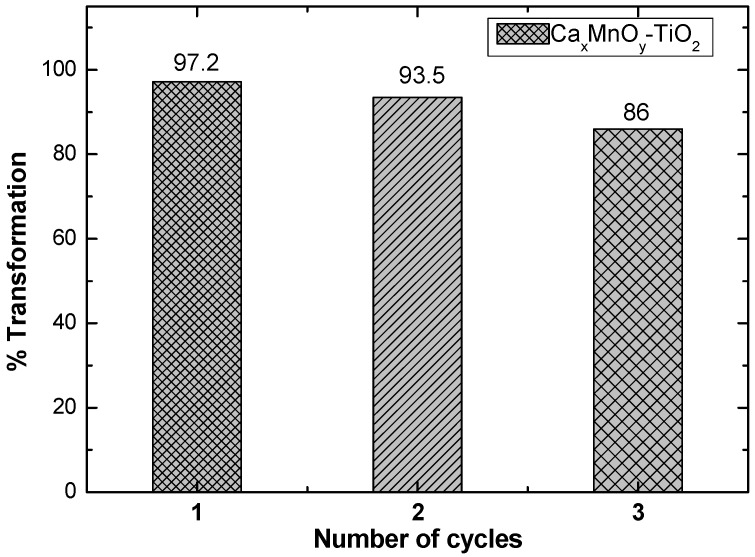
Catalytic recycling of Ca*_x_*MnO*_y_*-TiO_2_ in the transformation of Imazapyr.

The stability and reusability of Ca*_x_*MnO*_y_*-TiO_2_ photocatalyst was checked by using Ca*_x_*MnO*_y_*-TiO_2_ catalyst for three consecutive cycles, as shown in Figure 19, and in the third cycle, the percentage of degradation was found to be 86% due to the loss of some catalyst during washing and filtration. These initial results are highly encouraging to indicate the reusability of Ca*_x_*MnO*_y_*-TiO_2_ photocatalyst. However, further replicates are needed to confirm the chemical stability and reusability of the materials under various conditions for photocatalytic water treatment.

From these results, it seems clear that Ca*_x_*MnO*_y_* plays an important role in the enhancement of photocatalytic activity of TiO_2_. The formation of Ca*_x_*MnO*_y_*-TiO_2_ extends the spectral photocatalytic response into the visible region, indicating the possibility of the band-gap narrowing.

Ca*_x_*MnO*_y_* is present only on the surface of TiO_2_ since usually Mn-doped TiO_2_ are synthesized at higher temperatures (>600 °C) [13,27]. The reduction in the band-gap is proposed to be due to the surface modification of TiO_2_ via Ca*_x_*MnO*_y_*. The surface modification occurs via the formation of Ti-O-Mn bonds at the interface. XPS and TEM measurements support this hypothesis. The optical absorption of Ca*_x_*MnO*_y_*-TiO_2_ and TiO_2_ were measured from the valence band XPS and UV-Vis spectra. Ca*_x_*MnO*_y_*-TiO_2_ showed a shift in the energy of the valence band edge vs. Fermi level. The value of the band-edge for Ca*_x_*MnO*_y_*-TiO_2_ is 2.85 eV as compared to 2.62 eV for pure TiO_2_. The slight extension of the optical absorption may be attributed to the reduction in the effective band-gap.

Sayilgan et al. reported that manganese oxide facilitates the excited electron transfer to the surface and therefore reduces the chances of the recombination of photogenerated e^−^/h^+^ [47]. Ca*_x_*MnO*_y_* enhances the photocatalytic efficiency by acting as an electron acceptor to capture the electrons from TiO_2_ conduction band. This reduces the recombination efficiency of the photogenerated charge carriers. The photogenerated charge carriers reach to the surface for the generation of more reactive oxygen species which in turn can enhance the photocatalytic degradation of the pollutants.

**Figure 20:**
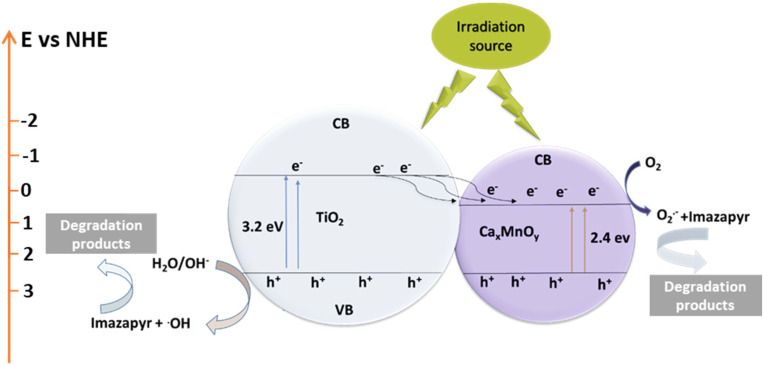
Band scheme for the enhancement in the photocatalytic activity of TiO_2_ by Ca*_x_*MnO*_y_* modification.

Surface modification of TiO_2_ by Ca*_x_*MnO*_y_* results in enhanced photocatalytic activity due to two reasons. First, the separation of the photogenerated charges at the heterojunction, which can be explained using a band scheme such as the one shown in Figure 20. Wherein, the photogenerated electrons from the conduction band of TiO_2_ transfer to the conduction band of Ca*_x_*MnO*_y_*. Ca*_x_*MnO*_y_* has a more positive conduction band potential which makes this process thermodynamically favourable. Under these circumstances, the holes in the valence band of TiO_2_ will generate HO^•^ radicals by oxidation of water and the e^−^ in the conduction band of Ca*_x_*MnO*_y_* will generate O_2_^•−^ by singe electron reduction of molecular O_2_. As the conduction band potential is more positive for Ca*_x_*MnO*_y_*, and considering that E^0^(O_2_/O_2_^•−^) = −0.33 V (vs. SHE) [48], it is less likely to be able to produce O_2_^•−^. This is supported by our BQ studies.

Hence, the two pathways will be open, one, based on HO^•^, as main ROS, faster, leading to highly hydroxylated compounds, and the other one, slower, based on O_2_^•−^ and leading first less hydroxylated compounds. This agrees with the observed products. The second reason is the reduction in the effective band-gap. As Ca*_x_*MnO*_y_* has a band gap in the visible region (2.4 eV, which corresponds to 516 nm), it generates electron hole pairs, giving the visible activity which is a fraction of the UV photocatalytic activity. 

## 4. Conclusions

Ca*_x_*MnO*_y_*-TiO_2_ heterostructures were prepared by the synthesis of birnessite by chemical co-precipitation method and subsequent mixing with commercial titanium dioxide by grinding, followed by annealing. As a result, Ca*_x_*MnO*_y_* layers are proposed to be encapsulating the TiO_2_ particles. Ca*_x_*MnO*_y_* contributed towards the enhancement of the photocatalytic activity of Ca*_x_*MnO*_y_*-TiO_2_ by three ways: extending the photon absorption into the visible range by slight band-gap narrowing, possibly improving charge transfer and lowering recombination of charge carriers, and by altering intermediate adsorption and degradation. The main intermediate products of photodegradation process of Imazapyr were examined. In the Ca*_x_*MnO*_y_*-TiO_2_ system, the concentration of intermediates continuously increased with irradiation time, to achieve a maximum at 180 min of irradiation, and decreased by more than half in the following hours, whereas a much slower decompositions of intermediates was observed using the unmodified TiO_2_. The enhanced photocatalytic activity was ascribed to the Ca*_x_*MnO*_y_* surface modification which adsorbs pollutant/intermediates with higher efficiency, thus improving their photocatalytic decomposition rate. The ROS responsible for the photocatalytic degradation of the pollutants was determined to be the hydroxyl radicals, as measured by scavenging studies.

## Figures and Tables

**Table 1:** The pH value, isoelectric point and zeta potential of pure TiO_2_ and Ca*_x_*MnO*_y_*-TiO_2_.

Catalyst	Zeta Potential (mV)	pH	Isoelectric Point
TiO_2_	26.3 ± 0.4	4.14	6.1 ± 0.2
Ca*_x_*MnO*_y_*-TiO_2_	37.7 ± 0.5	4.09	6.7 ± 0.2

**Table 2:** Photoproducts of Imazapyr degradation and their intensities identified by the positive and negative modes of MS-ESI.

Product Number	Formula	Molar Mass	MS-ESI		Intensity in the Presence of (TiO_2_) a.u.	Intensity in the Presence of (Ca*_x_*MnO*_y_*-TiO_2_) a.u.
			Positive Mode (M+H)^+^	Negative Mode (M-H)^−^		
1 (Imazapyr)	C_13_H_15_N_3_O_3_	261.281	262.14	260.45	20,696	14,760
2	C_12_H_15_N_3_O	217.272	218.7	216.2	3081	Nd.
3	C_12_H_15_N_3_O_2_	233.271	234.5	232.4	2096	Nd.
4	C_12_H_15_N_3_O_4_	265.269	266.42	264.13	1230	Nd.
5	C_11_H_17_N_3_O_3_	239.275	240.3	238	6051	Nd.
6	C_8_H_11_N_3_O_3_	197.194	198.2	196.5	5690	9340
7	C_10_H_9_N_3_O_4_	235.199	236.2	234.9	3120	948
8	C_9_H_7_N_3_O_3_	205.173	206.18	204.3	980	Nd.
9	C_7_H_5_NO_5_	183.119	Nf.	182.5	Nd.	586
10	C_7_H_5_NO_4_	167.120	Nf.	166.7	Nd.	Nd.
11	C_6_H_5_NO_3_	139.110	Nf.	138.48	1986	523
12	C_7_H_12_N_2_O_2_	156.185	Nf.	155.2	Nd.	396
13	C_3_H_4_N_2_O_2_	100.077	Nf.	99.8	428	291

## Supplementary Materials

Additional material available online at https://www.mdpi.com/2079-4991/10/5/896/s1. Figure S1. Average Spectra of 125 W Xenon irradiation source, Figure S2. (a) UV-Vis spectra and (b) absorbance vs concentration for Ca*_x_*MnO*_y_*-TiO_2_, Figure S3. XPS spectra of Ca*_x_*MnO*_y_*-TiO_2_ showing (a) Survey, (b) Ti 2p, (c) O 1s, (d) Ca 2p and (e) Mn 2p, Figure S4. Imazapyr photolysis under 125 W Xe under UV-Vis irradiation, Figure S5. Intensity of (M + H^+^) and (M + Na^+^) as a function of the concentration of Imazapyr solution, Figure S6: (a) HPLC calibration curve of phenol, (b) HPLC chromatograme for calibration of PhOH=Phenol, COH=Catechol, BQ=Benzoquinone and HQ=Hydroquinone, and (c) Shows the HPLC area vs phenol and intermediate concentration obtained from chromatograms of (b), Figure S7: (a) HPLC chromatograms obtained from TiO2, Ca*_x_*MnO*_y_*-TiO2 and P25 during the photocatalytic degradation of 1 mM phenol. In the figures: PhOH=Phenol, COH=Catechol, and BQ=Benzoquinone, Figure S8: (a) HPLC chromatograms obtained from TiO_2_, Ca*_x_*MnO*_y_*-TiO_2_ and P25 during the photocatalytic degradation of 78 µM imazapyr. In the figures: As shown in the circled regions of the figures, the areas under the intermediates is higher for P25 as compared to TiO_2_ and Ca*_x_*MnO*_y_*-TiO_2_.
