# Supplementary material for: Enhanced Photocatalytic Degradation of the Imidazolinone Herbicide Imazapyr upon UV/Vis Irradiation in the Presence of CaxMnOy-TiO2 Hetero-Nanostructures: Degradation Pathways and Reaction Intermediates
Source: Nanomaterials (Basel). 2020 May 8;10(5):896. doi: 10.3390/nano10050896 (PMC7279346; doi:10.3390/nano10050896)
Supplement: Supplementary file 1 [file nanomaterials-10-00896-s001.pdf]

Supplementary information (SI)

Photocatalytic degradation of imazapyr using Ca_xMnO_y-TiO_2 heterostructures under UV and visible irradiation: Identification of intermediates and investigation of new mechanism of reaction

Salma Bougarrani[1,*], Preetam K. Sharma[2,*], Jeremy W. J. Hamilton[2], Anukriti Singh[2], M. Canle[3,*], Mohammed El Azzouzi[1], J. Anthony Byrne[2]

[1] Laboratory of Spectroscopy, Molecular Modelling, Materials, Nanomaterials, Water and Environment, University Med V, Avenue Ibn Battouta, BP 1014, Agdal, Rabat, Morocco, salma.bougarrani@gmail.com

[2] NIBEC, Ulster University, Newtownabbey, BT37 0QB, United Kingdom, pk.sharma@ulster.ac.uk

[3] Chemical Reactivity and Photoreactivity Group, Department of Chemistry, Faculty of Sciences & CICA, University of A Coruña, E-15071 A Coruña, Spain, moises.canle@udc.es

*corresponding authors: S.B. (salma.bougarrani@gmail.com, +212660425050), PK.S. (pk.sharma@ulster.ac.uk , +44 2890368498)

SI 1. Irradiance and catalyst loading in the STR reactor

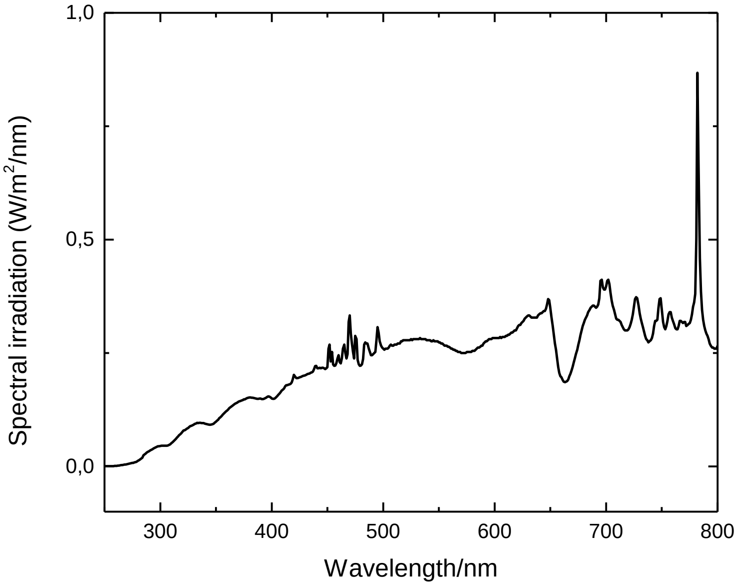

Figure. S1. Average Spectra of 125 W Xenon irradiation source

The irradiation source spectra were measured using Horiba Jobin Yvon spectroradiometer in 250-800 nm. The spectra at various points on the irradiated area were measured. The average spectra at the stirred tank reactor's surface is shown in figure S1.

The optimal loading of the catalyst was determined by measuring the absorbance in the UV range (280-400). The UV absorption by the catalyst was used to determine the concentration of the catalyst required to absorb 99% of the photon in the reactor. For 200 ml volume (photocatalytic test volume), the path length of the photons in the reactor is around 6 cm. So, we determined the concentration of the catalyst required for 99% absorption in 6 cm path length.

The UV-Vis absorbance spectra for various concentrations of Ca_xMnO_y-TiO_2 is shown in figure S2(a). The absorption spectra were superimposed on the Xe lamp spectra in 280-400 nm range ($A_{solar}=\sum A_y S_y/\sum S_y$) to obtain the absorbance with respect to Ca_xMnO_y-TiO_2 concentration (Figure S2(b)). Figure S2(b) was used to determine the molar extinction coefficient (ε). The Beer-Lambert law was used to determine the concentration corresponding to absorption of 99% (A=2) of the photons in 6 cm path length.

$$A=\varepsilon\cdot C\cdot l;\ C= A/(l\cdot\varepsilon) =30.6 \text{ mg/L} \qquad \text{--(equation S1)}$$

Here, l is 6 cm (reactor path length) and molar extinction coefficient (ε) is 0.01077 $M^{-1}\cdot cm^{-1}$. The values of A, ε, and l were used to determine the optimal loading which comes out as 30.6 $mg\cdot L^{-1}$.

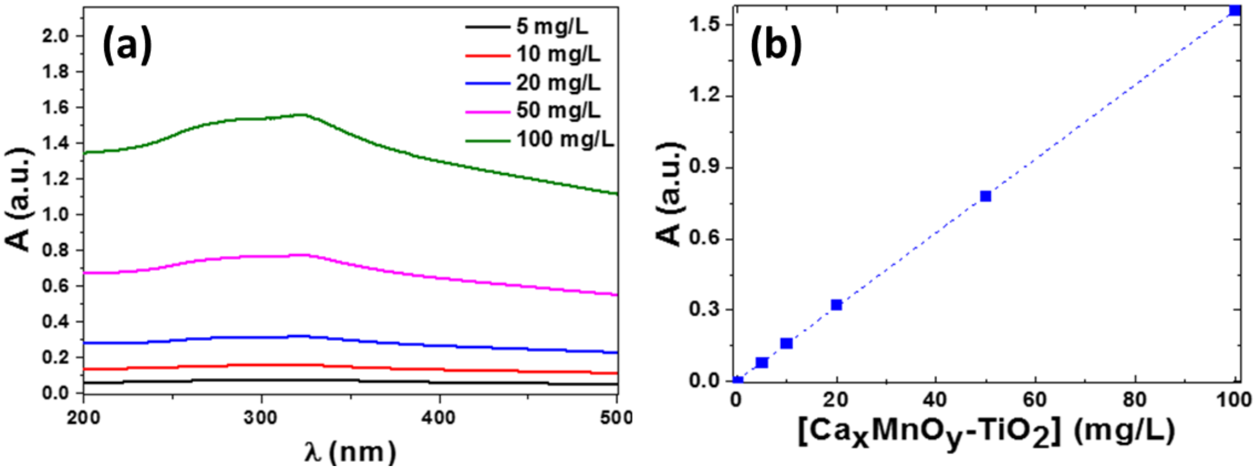

Figure. S2. (a) UV-Vis spectra and (b) absorbance vs concentration for Ca_xMnO_y-TiO_2

SI 2. Characterization of TiO_2 and Ca_xMnO_y-TiO_2 materials

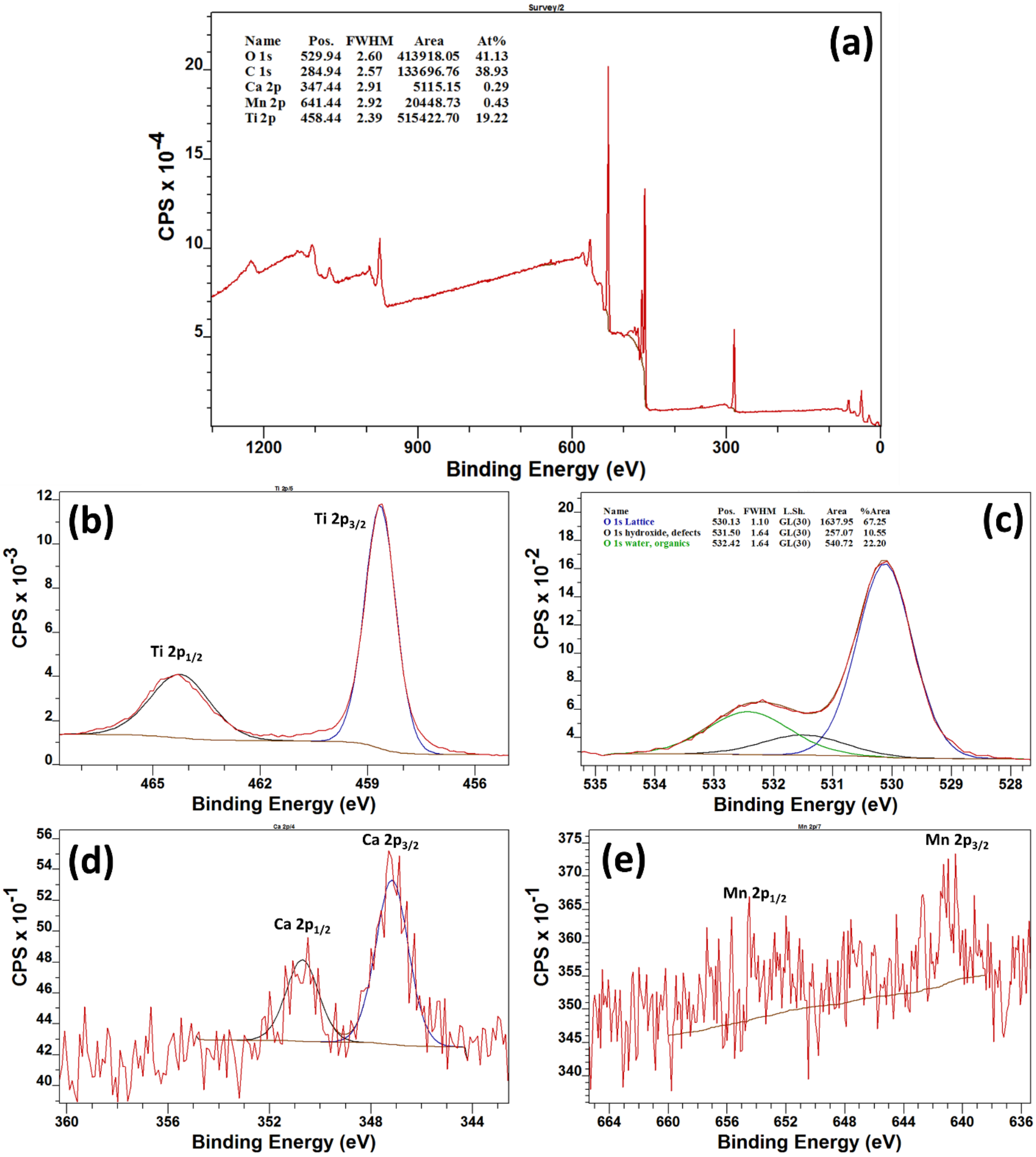

Figure. S3. XPS spectra of Ca_xMnO_y-TiO_2 showing (a) Survey, (b) Ti 2p, (c) O 1s, (d) Ca 2p and (e) Mn 2p.

SI 3. Controls for the photocatalytic degradation experiments

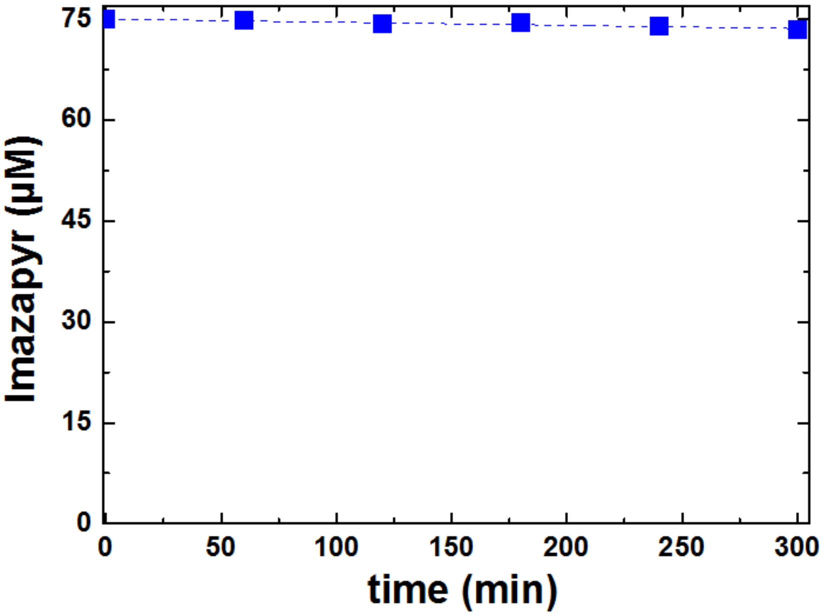

Figure. S4. Imazapyr photolysis under 125 W Xe under UV-Vis irradiation.

The photolysis experiments for imazapyr were performed in the stirred tank reactor under Xe UV-Vis irradiation (125 W). The samples were collected every hour and were analysed in HPLC. Over the 5 h irradiation, the imazapyr concentration reduces by 2% as shown in figure S5. The photolysis rate is much lower than the photocatalysis rate.

The dark adsorption measurements on TiO_2 and Ca_xMnO_y-TiO_2 were performed at the start of corresponding experiment for 1 h and the adsorbed amount of imazapyr and phenol were within the noise range.

SI 4. ESI-MS intensity vs concentration linearity

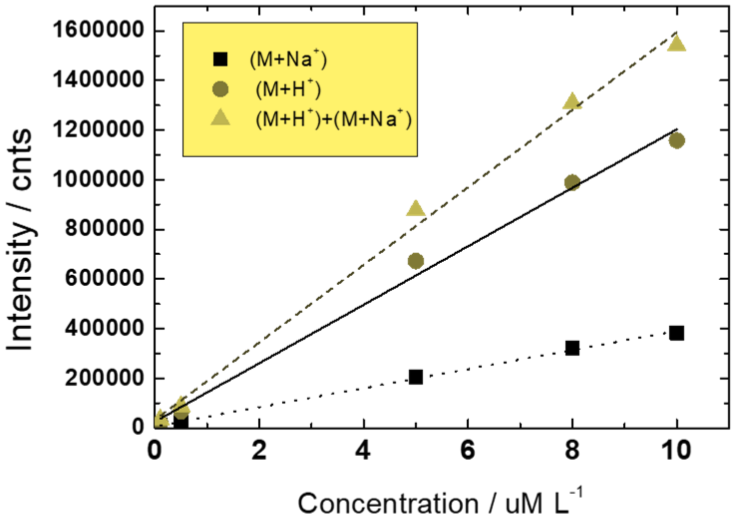

Figure. S5. Intensity of (M+H+) and (M+Na+) as a function of the concentration of Imazapyr solution

Specificity, linearity and recovery studies were conducted by injecting of different known concentrations of linearity solutions 10, 1, 0.1, 0.05 and 0.01 μg.L^{-1}. The solutions were prepared by serial dilution. The changes in concentrations of imazapyr were concluded from intensity of the peaks.

SI 5. HPLC calibration and measurements for the photocatalytic degradation experiments

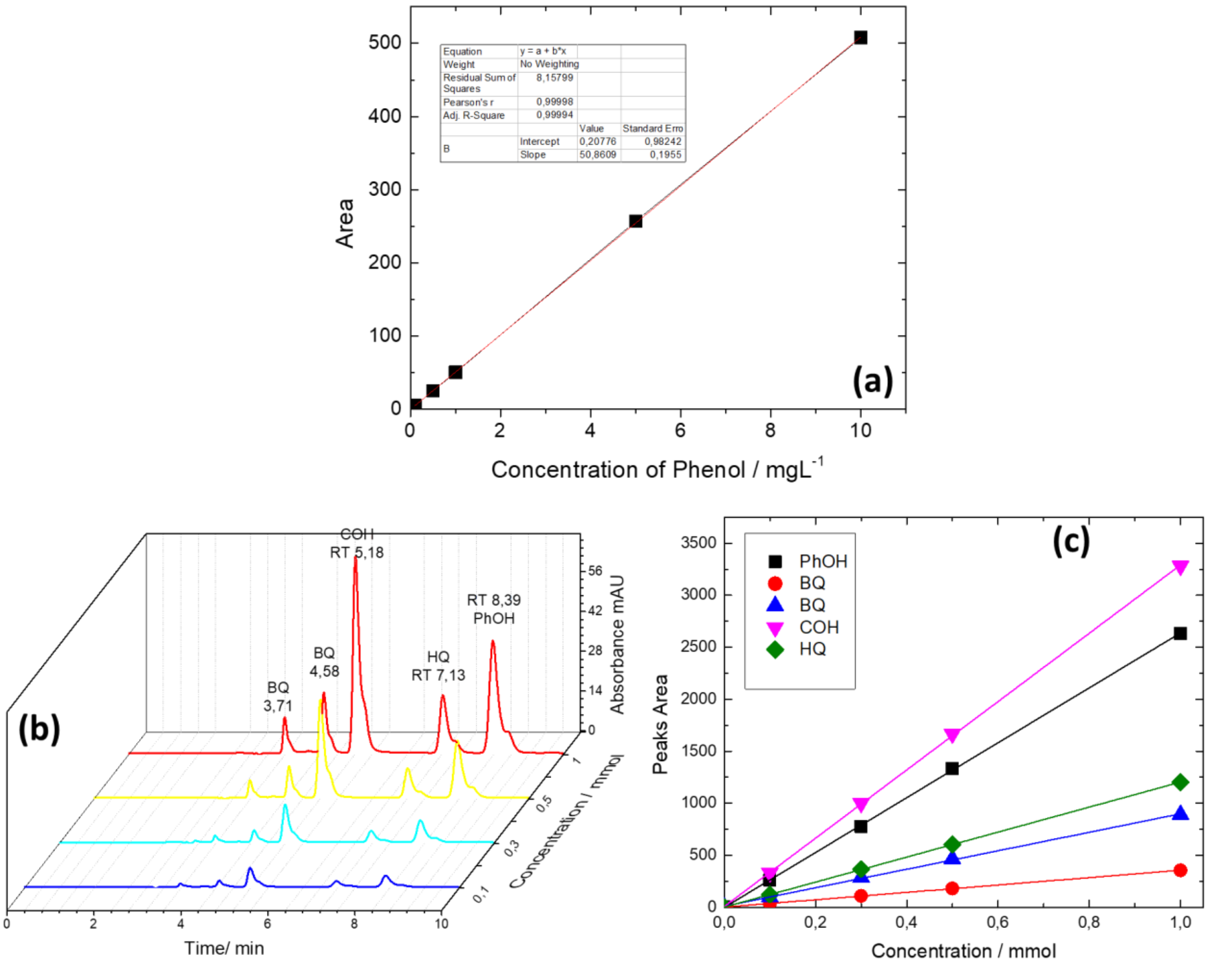

Figure S6: (a) HPLC calibration curve of phenol, (b) HPLC chromatograme for calibration of PhOH=Phenol, COH=Catechol, BQ=Benzoquinone and HQ= Hydroquinone, and (c) Shows the HPLC area vs phenol and intermediate concentration obtained from chromatograms of (b).

Figure 6 shows the represeentative HPLC calibration curve for phenol and intermediates.

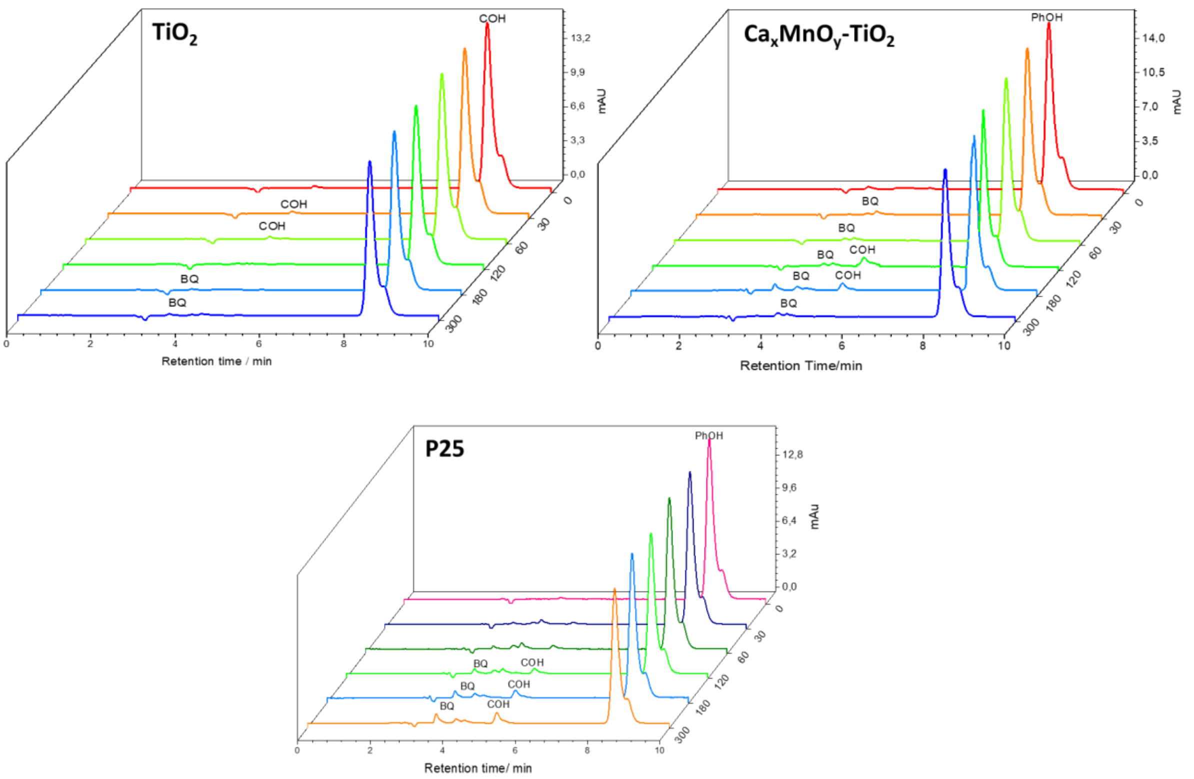

Figure S7: (a) HPLC chromatograms obtained from TiO2, CaxMnOy-TiO2 and P25 during the photocatalytic degradation of 1 mM phenol. In the figures: PhOH=Phenol, COH=Catechol, and BQ=Benzoquinone.

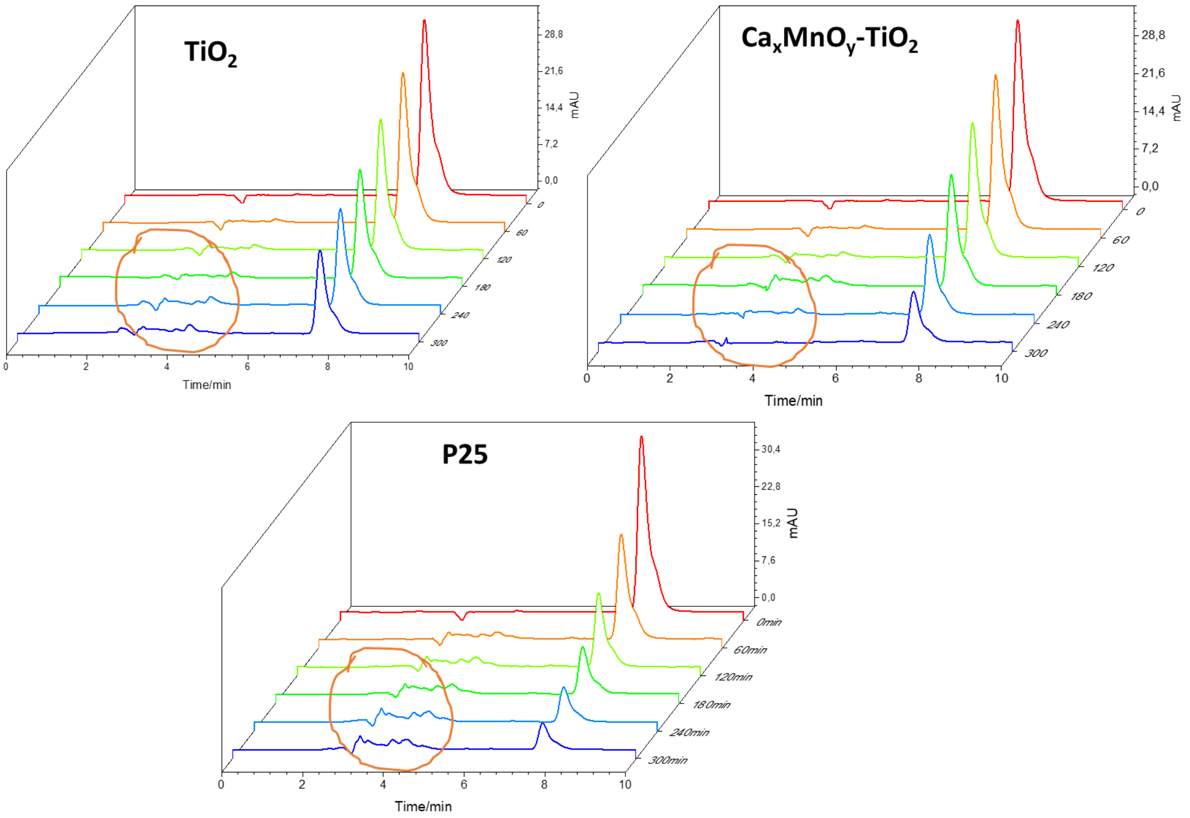

Figure S8: (a) HPLC chromatograms obtained from TiO_2, Ca_xMnO_y-TiO_2 and P25 during the photocatalytic degradation of 78 μM imazapyr. In the figures: As shown in the circled regions of the figures, the areas under the intermediates is higher for P25 as compared to TiO_2 and Ca_xMnO_y-TiO_2.
